# New Haven Dental Society

**Published:** 1864-07

**Authors:** C. Longstreet Smith

**Affiliations:** New Haven, Ct.


### NEW HAVEN DENTAL SOCIETY.

The dentists of New Haven met pursuant to adjournment at informal meeting, at the office of Dr. Samuel Mallett.

Dr. John T. Metcalf was appointed as chairman.

After reading the minutes of previous meeting, and being approved, the Committee on Constitution and By-Laws reported, and report accepted, and adopted as the basis of organization for the New Haven Dental Society.

The following gentlemen became members on signing the aforesaid Constitution and By-Laws: Drs. Samuel Mallett, Jos. D. Riggs, Isaac Woolworth, Jos. H. Smith, Aug. B. Smith, John T. Metcalf, James A. Dibble, Elias Strong, J. H. Reed, ——— Boutwell, H. I. Stevens, C. Longstreet Smith.

The Society then proceeded to an election of officers, resulting as follows; Drs. J. T. Metcalf, as President; S. Mallett as Vice-President; C. Longstreet Smith as Secretary; A. B. Smith as Treasurer; J. D. Riggs, as Librarian.

The assumption of office by the President elect elicited extended remarks "apropos" to our profession in general, and to this embryonic Society in particular.

The regular meetings are held on the evenings of the first Wednesday in each month.

The mutual good that has accrued thus far is incalculable, fully compensating for all the anxiety and trouble attending an incipient organization.

At the regular meeting in June, among other business transacted, was the appointing of Drs. J. T. Metcalf, E. Strong and C. Longstreet Smith to represent this Society in the American Association at Niagara in July ensuing.

C. LONGSTREET SMITH, *Secretary.*

*New Haven, Ct., June* 18, 1864.

